# Bioavailability of Methionine-Coated Zinc Nanoparticles as a Dietary Supplement Leads to Improved Performance and Bone Strength in Broiler Chicken Production

**DOI:** 10.3390/ani10091482

**Published:** 2020-08-24

**Authors:** Ashraf Alkhtib, Dawn Scholey, Nicholas Carter, Gareth W.V. Cave, Belal I. Hanafy, Siani R.J. Kempster, Subbareddy Mekapothula, Eve T. Roxborough, Emily J. Burton

**Affiliations:** 1 School of Animal, Rural and Environmental Sciences, Nottingham Trent University, Nottingham NG25 0QF, UK; ashraf.alkhtib@ntu.ac.uk (A.A.); dawn.scholey@ntu.ac.uk (D.S.); 2 School of Science and Technology, Nottingham Trent University, Nottingham NG11 8NS, UK; nicholas.carter@ntu.ac.uk (N.C.); belal.hanafy@ntu.ac.uk (B.I.H.); siani.kempster2015@my.ntu.ac.uk (S.R.J.K.); subba.mekapothula@ntu.ac.uk (S.M.); e.roxborough@lancaster.ac.uk (E.T.R.)

**Keywords:** broiler, zinc supplementation, tibia, nanoparticle

## Abstract

**Simple Summary:**

Nanotechnology is becoming widely accepted and being adapted for use in different fields. In the animal feed sector, there is potential for delivery of minerals using a nano format to increase bioavailability and digestibility. Broiler chickens and zinc were chosen as a model for examining nano mineral delivery. The trial showed that amino acid coated zinc improved bird growth and feed consumption and also improved digestibility of zinc. A further benefit was an improvement in leg strength of the supplemented birds. This suggests that coated nano delivery of minerals can improve their bioavailability with implications for production of mineral supplements in the animal feed industry.

**Abstract:**

Recently, nanotechnology has been widely adopted in many fields. The goal of this study was to evaluate the potential for amino acid coated nano minerals as a supplement in broiler feed. Zinc was selected as a model mineral for this test and supplementation of nano zinc, both coated and uncoated was compared with organic and inorganic commercial forms of zinc. A total of 48 pens (8 birds each) were assigned to one of the following dietary treatments: Control, methionine-Zinc chelate (M-Zn), nano zinc oxide (Nano-ZnO), and methionine coated nano zinc oxide (M-Nano-ZnO). All experimental diets were formulated with the same total zinc, methionine, protein, and energy content with just the zinc source as a variable. Bird weight, feed intake and feed conversion ratios were recorded weekly, with three birds culled (sacrificed) at day 21 and day 35 for sampling measures. Ileal digestibility of zinc was determined at day 21 and day 35 using titanium dioxide as an inert marker. Blood serum, liver and spleen samples were collected at day 21 and day 35 and analysed for zinc content via inductively coupled plasma mass spectrometry (ICP-MS). Tibia strength and morphometrics were measured from both legs of three birds per pen at day 21 and day 35. The study was conducted at Nottingham Trent University Poultry Unit, UK. The novel method of producing nano minerals coated with amino acids was successfully tested with zinc and material produced to test in the feeding study. Methionine coated nano zinc oxide supplementation significantly improved bird weight gain and the increased feed intake of broilers compared to an inorganic zinc form. Ileal digestibility was also improved with this methionine-nano zinc. Moreover, this supplementation improved the tibia strength of broilers at the age of 21 days, though this was not observed at day 35. Therefore, M-Nano-ZnO could be used to supplement broilers to improve both performance and digestibility with a limited positive impact on bone strength. The results of the current study suggest that the amino acid coating of nano minerals can improve the digestibility of minerals which may have further implications for the field of mineral nutrition in animal feeds.

## 1. Introduction

Recently, nanotechnology has been widely adopted as a new trend in many fields. There is potential for nanoparticles to effectively deliver essential trace minerals in animal feed, with the potential for increased mineral bioavailability. This improved availability can be attributed to both the small size and large surface area to volume ratio of these particles [1,2,3]. The incorporation of a functionalised coating such as an amino acid can improve the stability of nanoparticles in solution and assist their uptake [4]. A novel process has been developed for producing coated nanoparticle minerals which may have an application in the delivery of supplementary minerals. For initial studies to quantify nanoparticle bioavailability of minerals in broilers, zinc was chosen as a mineral which requires supplementation, but where tolerance in the birds is high.

Zinc is an essential trace element for all living species [5]. In its dominant, + 2 oxidation state, zinc is integral to a number of biochemical pathways as a catalytic or regulatory cofactor, and also has structural roles in many other functional proteins [6]. Therefore, zinc is necessary for normal growth and maintenance, restoring damaged tissues [7] and is vital for the development of bones and feathers [8,9].

Many plant and animal sourced poultry feeds contain zinc, but they also commonly contain substantial quantities of phytate. Each phytate binds very strongly to six zinc (II) ions, preventing its absorption in the gastrointestinal tract [10]. This lowers the bioavailability of zinc in these diets to below requirements for healthy growing poultry [11]. Zinc deficiency in poultry has been shown to cause slow growth, shortened and thickened legs with an enlarged hock and frizzled feathers [12]. To negate these effects, poultry diets are routinely supplemented with additional zinc. Current published values for the zinc requirement of broilers per Kg of feed are advised at between 40 and 60 mg/Kg. However, recommendations from the European Union feed industry are far higher: 70–120 mg/Kg diet [13].

Zinc is tolerated by animals at relatively high dietary amounts. If the requirement for zinc is markedly exceeded, additional zinc is not absorbed but passes through the gastrointestinal tract and is excreted with litter. The maximum allowable levels of zinc in animal diets has received recent attention due to concerns over the potential environmental impact of zinc from nutritional sources [14]. While the current focus relating to poultry production is nitrates and phosphates, there are a number of territories in Europe facing reduced crop yields due to excessive soil zinc and copper levels; although, currently these are mainly associated with pollutants from steel galvanisation industries [15]. The implications of soil zinc contamination from poultry manure on European food security has led the European Feed Standards Agency (EFSA) to lower the maximum permitted inclusion level of zinc in poultry diets from 250 mg/Kg to 150 mg/Kg diet [13]. Although the prescribed 150 mg/Kg diet is more than double the level required to avoid zinc deficiency symptoms in broilers, dietary zinc currently has an additional and crucial role in the normal development of the immune and skeletal systems [6]. Thus, there is a need to adopt new forms of zinc supplements that are highly bioavailable, in order that the wellbeing benefits of high zinc status may be achieved without excessive zinc excretion into the environment.

Several zinc substrates are currently used as nutritional feed additives; these are commonly categorized as inorganic (such as zinc acetate, zinc oxide and zinc sulphate) or organic (including amino acid zinc chelates of glycine, lysine or methionine, and their sulphate and hydrate analogues) [16]. Amino acid chelated forms of zinc are commonly reported to enhance the bioavailability of zinc as these provide considerable protection from formation of indigestible complexes with phytic acid [17]. Recent research has suggested there is also potential for these forms of zinc in terms of gut health and development [18]. Zinc oxide nanoparticles are typically formed via one of two synthetic routes: a vapour phase reaction between zinc metal vapour and oxygen or a co-precipitation reaction between a zinc metal salt and base [19]. The wet chemical route allows the production of a diverse, yet highly tuneable, range of particle sizes and morphologies with the ability to functionalised the surface of the particles to alter their properties in suspension [20,21]. Precise control of the size and morphology of nanoparticles can be attained by using novel reactor systems such as the spinning disc reactor [22]. These features of the spinning disc reactors (SDR) process enable the increased production of narrow ranged nano-particles (NP) that can be tailor-synthesised to specific requirements using coprecipitation and sol gel methods. This enables new materials to be developed which require mass produced nanomaterials, applying a nanotechnology ethos of improved material efficiency into areas such as agriculture. The use of silver NP has been successfully transferred from medical applications as an antifungal and anti-bacterial agent [23], to pathogen control in the prevention of fungal diseases [24] like powdery mildew [25]. In the spinning disc reactor, two separate solutions of a zinc metal salt and base are pumped onto a spinning disc, the two solutions rapidly mix on the surface of the disc and nanoparticles are precipitated. By precisely controlling the flow rate, disc speed, precursor concentration and reaction temperature, the size and morphology of nanoparticles can be tailored [22].

To date, no studies have reported on the effect of methionine-coated nanoparticle zinc in broilers. Thus, the aim of the current study is to determine the effect of methionine coated nano zinc oxide on performance, zinc digestibility and absorption. In addition, due to the role of zinc in bone health, the effect of nanoparticle zinc supplementation on bone traits was observed.

## 2. Materials and Methods

### 2.1. Zinc Oxide Nanoparticle Preparation

Aqueous sodium hydroxide (1 M) was added dropwise to a stirred, heated aqueous solution of a zinc chloride precursor. The mixture was stirred for 30 min, the resulting white precipitate was then cooled in an ice bath and collected in vacuo using a sintered crucible (grade 3). The precipitate was washed three times with ice-cold deionised water and dried in an oven overnight. Prepared zinc oxide nanoparticles (Nano-ZnO) were ground using a pestle and mortar then stored in an air-tight container.

Coating the Nano-ZnO with L-methionine was achieved by grinding the dry zinc oxide nanoparticles (100.2 g, 25 nm) with L-methionine (200.0 g, Glentham Life Sciences, London, UK) in a colloidal mixer, producing a eutectic melt that hardened to a white solid (ca. 15 min) which was then ground with a pestle and mortar to a fine powder. The final M-Nano-ZnO product was stored in an air-tight container under a nitrogen atmosphere until diet manufacture.

### 2.2. Nanoparticle Size Determination

Nanoparticle size was determined by scanning electron microscopy (SEM) and transmission electron microscopy (TEM) imaging. SEM images of Nano-ZnO and M-Nano-ZnO powder samples were collected using a JSM-7100F Field Emission Scanning Electron Microscope (Jeol, Tokyo, Japan) operating at an accelerating voltage of 5 kV, with low probe current (1 or 2) and a working distance of 4 or 6 mm as required. TEM images were collected using a JEM 2100 Plus with LaB6 (Jeol, Tokyo, Japan). Samples were then prepared for TEM analysis as follows: ground nanoparticles (1 mg) were added to deionised water (10 mL). The sample was sonicated for 30 min with occasional shaking. Parafilm was placed on filter paper in a petri-dish. A copper TEM grid was placed on top of the parafilm and a 4 µL drop of the relevant sample was placed onto the TEM grid. The lid of the petri-dish was replaced, and the sample was left for 24 h to allow the deionised water to evaporate prior to analysis.

### 2.3. Dietary Treatments 

A two-phase diet was formulated (Table 1) on a wheat-soya bean meal base to meet the nutrient requirements of the age and strain of birds in all key nutrients other than zinc, which was added separately to each dietary treatment as described below. Diets were manufactured in house from a basal, zinc-free mash (Target Feeds Ltd., Shropshire, UK) with titanium dioxide included in all diets at 5 g/Kg diet as an inert digestibility marker. All diets received 50 g of added zinc per ton of feed, as one of four forms of zinc: Control (feed grade zinc oxide), M-Zn (methionine-zinc chelate, Pancasma B-Traxim), Nano-ZnO (nano zinc oxide. 25 nm) and M-Nano-ZnO (methionine coated, nano zinc oxide, 25 nm). Zinc oxide was chosen as a negative control diet due to the comparatively poor bioavailability of zinc in this inorganic format. As M-Nano-ZnO contained a 1:2 ratio of zinc to methionine coating, all other diets were also supplied with additional methionine at a 1:2 zinc to methionine ratio based on the volume of zinc supplement added per ton of feed.

### 2.4. Birds And Husbandry 

A total of 384 Ross 308 male broilers (to reduce variability) were obtained from a commercial hatchery on day of hatch. Birds were individually weighed before being randomly assigned to 48 pens (80 * 80 cm; 0.64 m^2^ pens; 8 birds per pen, 12.5 kg/m^2^ stocking density at day 21). The experimental birds were bedded on clean wood shavings. Each experimental chick was vaccinated against Marek’s disease, infectious bronchitis and Newcastle disease at the hatchery. Pens were randomly allotted to the four dietary treatments (12 pens/treatment) as follows, Control, M-Zn (methionine-Zinc chelate), Nano-ZnO (nano zinc oxide) and M-Nano-ZnO (methionine coated nano zinc oxide).

The initial temperature of the room was set to 31 °C (50–60% relative humidity) on day 1 then reduced gradually by 1 °C per day, until 21 °C was reached which was maintained for the remainder of the trial. The lighting regimen in the room started as 23 h light on day 1, with darkness increasing by one hour/day until 6 h of darkness was reached. This was maintained throughout the remainder of the study. Bird weight/pen, feed intake and feed conversion ratio were recorded weekly across the study and mortality was recorded daily and any birds culled or found dead were weighed. Mortality over the 35 days was 6.5% split evenly between treatments, including 2.8% mortality in the first week from congenital issues and 1.8% from birds culled as runts at day 21. Institutional and UK national NC3R ARRIVE guidelines for the care, use and reporting of animals in research [26] were followed during the study and all experimental procedures were approved by Nottingham Trent University’s animal ethics review committee (internal code ARE887).

Three birds/pen were randomly selected on day 21 post hatch and euthanised, and the remaining birds were euthanised at the end of the study on day 35. Euthanasia was carried out by cervical dislocation without prior stunning. Tibia, ileal digesta, blood, liver and spleen were collected from three birds per pen at both day 21 and day 35. Blood samples were collected by cardiac puncture immediately post-mortem and pooled from the three birds into a tube. Blood was allowed to clot for 60 min before centrifugation at 3000 rpm for 5 min and serum collected and frozen at −20 °C. Ileum digesta contents from three birds were collected by gentle digital pressure into one pot pen and stored at −20 °C prior to freeze drying. Once freeze dried, the samples were finely ground with a pestle and mortar. Liver and spleen were collected from three birds per pen, at both day 21 and day 35 and stored at −20 °C prior to analysis. Tibias were removed between the tibial–tarsal joint and the tibial–femoral joint, and defleshed of muscle and tissue and stored at −20 °C for further analysis.

### 2.5. Diet Analysis

Nitrogen content of the diets was determined using a combustion analyser (Dumatherm N Pro, Gerhardt Analytical Systems, Königswinter, Germany) then multiplied by 6.25 to derive crude protein content. Extractable fat content, dry matter and were was analysed according to the method described in [27] (2003.05, 930.15 and 942.05, respectively). Following an aqua regia digestion step (AOAC (Association of Official Agricultural Chemists) 985.01), calcium, phosphorus and zinc content were analysed using an inductively coupled plasma optical emission spectrometer (ICP-OES, Perkin Elmer Avio200 Model – 725 radial view; Waltham, MA, USA) while gross energy was determined using an adiabatic bomb calorimeter (Parr, Moline, IL, USA) with benzoic acid as a calibration standard. Nutrient digestibility was calculated via an inert marker by determining titanium dioxide in diets and digesta samples by acid digestion and UV-spectroscopy [28].

### 2.6. Blood And Tissue Zinc Measures 

The levels of zinc were determined by inductively coupled plasma mass spectrometry (ICP-MS) using a PerkinElmer NexION 1000 ICP-MS instrument (Waltham, MA, USA). Analysis was performed on chicken liver, serum, digesta and spleen samples with standard calibration from Certipur^®^ ICP (Merck, Watford, UK) Single-Element standards of zinc.

To determine serum zinc concentration, 0.4 mL of the serum was added to 4.6 mL of concentrated nitric acid in a 15 mL conical centrifuge tube. The samples were then diluted appropriately for ICP-MS analysis. For the liver, spleen and digesta samples, approximately 0.5 g of freeze-dried tissue was added to a 10 mL conical centrifuge tube containing approximately 8 mL of concentrated nitric acid. The mixture was sonicated for approximately 2 h, the volume of concentrated nitric acid was made up to 10 mL in a volumetric flask and the samples were filtered with a 0.2 µm membrane syringe filter before being diluted ready for ICP-MS analysis. Standards were prepared in dilute ultrapure nitric acid (1%) ranging between 0.2, 2, 20, 50, 100, 250, 500, 750 and 1000 ppb. Data were obtained as ppb and converted to mg/Kg for liver, spleen and digesta and to µg/dL for serum.

The ground digesta samples were analysed for titanium dioxide content by the method described in [29]. Apparent ileal zinc digestibility coefficients (COD) were obtained using the following equation (all measures in g/Kg Dry matter):

(nutrient/TiO_2_)_diet_ − (nutrient/TiO_2_)_ileum digesta_/(nutrient/TiO_2_)_diet_ (1)

Digested zinc was calculated by multiplying the COD by the amount of zinc in the diets.

### 2.7. Tibia Measurements

Tibia measures were made on three birds per pen at both day 21 and day 35. All measures were collected blind (without treatment identification) by the same operators. Width, length and weight of left and right tibiae of individual birds were recorded and then averaged. Length was measured from the most extreme points of the tibia, with cartilage caps included using digital calipers. Width was measured at the marked mid-point of each bone (premeasured). Orientation of the bones was consistent for both measures (nodules down and on the left of the operator) and data were collected by one operator to improve consistency. Bone strength of both the tibiae were analysed using a TA.XT plus texture analyser (Stable Microsystems, Guildford, UK) set up with a 50 kg load cell and 3 point-bend fixture [30]. The texture analyser was set to measure force in compression; test speed was set at 1 mm/s, and trigger force was set at 7 g (0.069 N). The defleshed bone was placed on the fixtures, a test was run and the peak force in Newtons was recorded. To standardise measurements of strength, the bone was always broken at the mid-point previously marked for width measurement. The bones were placed on the texture analyser in the same orientation and plane - bottom to the left and head to the right, with the nodules facing down. The 3-point bend rig on the texture analyser was adjusted for length of bone each time.

### 2.8. Statistical Analysis

The experimental pens were blocked into 12 blocks (1 experimental diet each) to account for the variability in temperature and humidity in the room. Data were analysed using the following model:

Y_(ij)_ = µ + TRT_(i)_ + block_(j)_ + Ɛ_(ij)_

where Y is the response variable, µ is the overall mean, TRT is the effect of treatment I, block is the effect of block j and Ɛ is the residual. Means were separated using least significant difference method at a 0.05 level of probability. All data analyses were performed using R [31].

## 3. Results

**Figure 1:**
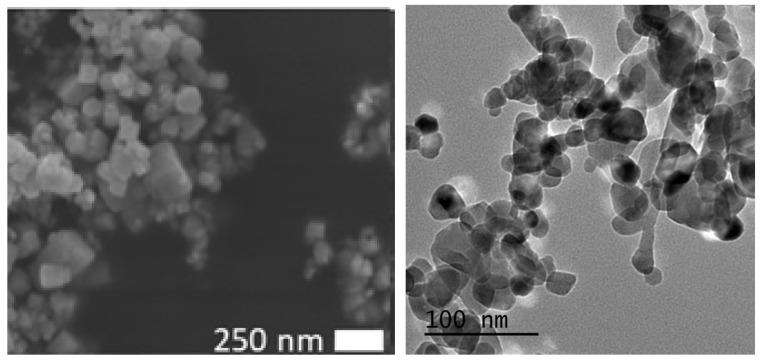
Scanning electron microscopy (**left**) and transmission electron microscopy (**right**) images of methionine coated nano zinc (M-Nano-ZnO; 50,000× magnification).

The sizes of the zinc nanoparticles were determined by Scanning electron microscopy (SEM) and Transmission electron microscopy (TEM) (see Figure 1). Characteristic zinc oxide FT-IR stretches were observed at 695 cm^−1^ and 911 cm^−1^. Dynamic light scattering data of coated zinc nano’s in deionised water at room temperature: average size 11.2 nm diameter with a zeta potential of −3.85 mV.

Chemical composition and gross energy content of the basal diets are presented in Table 1. Total measured zinc contents of starter diets were 83 mg/kg Control, 99 mg/kg M-Zn, 73 mg/kg Nano-ZnO and 86 mg/kg M-Nano-ZnO and the total measured zinc contents of grower diets were 91 mg/kg Control, 81 mg/kg M-Zn, 59 mg/kg Nano-ZnO and 72 mg/kg M-Nano-ZnO. Table 2 shows the ileal zinc digestibility of broilers at day 21 and day 35. M-Nano-Zn had a significantly higher COD at day 21 compared with the control and Nano-Zn, but it was not significantly different to M-Zn. The M-Zn diet contained slightly higher measured zinc so the digested zinc in this diet was correspondingly higher compared with M-Nano-Zn. At day 35, M-Nano-Zn again had the highest COD compared to M-Zn and Nano-Zn, though it was not significantly higher than the control. The dietary zinc content of Nano-Zn was lower for this phase, resulting in a lower level of digested zinc in this diet.

Table 3 presents the growth performance and feed conversion ratio of the experimental birds by phase and overall. Bird weight gain (BWG) in the starter phase (days 0–21) was significantly improved for the M-Nano-Zn diet compared with the other diets and birds fed the control diet were significantly lighter for this phase compared with all other treatments. In the grower period, (days 21–35) there was no significant difference in BWG or Feed Conversion Ratio (FCR), but a trend that M-Nano-Zn had higher feed intake (FI) than the control or M-Zn was observed. Overall performance (days 0–35) showed that birds were heavier when fed the Nano Zn diet compared with the control, and there was a trend that the values for M-Nano-Zn were higher than for M-Zn (*p* = 0.076). FI was also higher for the M-Nano-Zn compared with all other diets, and there was no effect on FCR.

The effect of zinc type on tibia strength and morphometrics is presented in Table 4. Tibias were significantly longer for M-Nano-Zn and M-Zn compared with the control and Nano-Zn at day 21. A similar pattern was seen for weight and width measures, Tibia breaking strength was significantly higher for M-Zn compared with the control and Nano-Zn, but there was no difference between M-Nano-Zn and M-Zn at day 21. By day 35, there was little difference in the tibial measurements seen between treatments. The only significant effect at day 35 was on tibia length, which was increased for Nano-Zn compared with the control and M-Zn and for M-Nano-Zn compared with the control.

The effect of zinc type on zinc content of serum, liver and spleen is presented in Table 5. The dietary treatment did not significantly affect zinc content of serum, liver or spleen at either day 21 or day 35.

## 4. Discussion

This study investigated the potential for nano-delivery of amino acid coated minerals for improving bioavailability in vivo, using zinc as a model for the delivery format. As such, the M-Nano-Zn product was compared with a low cost inorganic source of Zn (zinc oxide; Control), expected to have low availability and with the industry standard chelated zinc M-Zn, expected to have high bioavailability. To illustrate if the nano material alone is bioavailable without amino acid coating, nano zinc oxide was also fed to the birds. Throughout the starter period and overall, the methionine-coated nano zinc supplemented diets improved the weight gain of broilers in the current study compared to the control. Previously reported studies using diets without phytase are likely to demonstrate enhanced differences between inorganic and organic forms of zinc. Control diets for the current study contained phytase to minimise interaction between dietary phytate and zinc so that findings may be considered relevant to current commercial diets. Additionally, birds fed methionine-coated nano zinc oxide showed weekly weight gains 2–5% higher than those fed the non-coated nano zinc oxide, but this improvement was only significant at day 35. This degree of improvement in weight is relevant in a commercial setting but requires verifying in a larger scale study with increased statistical power. In the first 21 days of the study, the chelated zinc and uncoated nano zinc also significantly improved weight gain, but this improvement was no longer significant towards the end of the study. The bodyweight of the control birds was between 87% and 94% of Aviagen breed targets at day 21 and d35 respectively, but the M-Nano-Zn diet produced bodyweights close to breed targets (within 10 g), even though the diets were mash format and would be expected to produce poorer performance. Previous studies have also reported inconsistent effects of chelated form performance. In the current study, it is possible that some degree of heat stress affected performance of birds towards the end of the study. Daytime temperatures within the UK were the highest ever recorded [32] during the 28th and 35th day of the study, which is likely to have affected larger birds (specifically those on diets which showed improved performance in days 1–21 of the study) due to their increased feed intake and decreased ability to dissipate heat [33]. Specifically, days 23 and 24 recorded average room temperatures of 28 °C and a maximum temperature of 32 °C. Differences in pathogen challenge and oxidative status also contribute to varied response to zinc supplementation, which may explain the lack of consensus between previously reported studies [34].

The driving force behind the observed increase in body weight gain associated with feeding M-Nano-ZnO appears to be a combination of improved dietary nutrient utilisation (manifested as significantly increased coefficients of zinc digestibility) and increased feed intake. The improvement in feed intake due to M-Nano-ZnO is in agreement with [35] who reported positive effects of zinc supplementation on the appetite of broilers. However, a number of other mechanisms may be contributing to the improved growth as other studies have reported altered growth hormone production and enhanced capacity for immune response, alongside improved growth [3,36]. Zinc digestibility was significantly improved in terms of digestibility coefficients for the M-Nano-Zn, which suggests that the coated nano delivery is producing an improvement in bioavailability. The high levels of zinc digestibility associated with coated nano-delivery suggest that the health concerns sometimes associated with insoluble nanoparticles are not relevant to this form of nanoparticle [37].

Serum zinc levels were not significantly different between treatments, but previous studies have also reported that increased bioavailability does not necessarily induce differences in zinc serum levels [38,39]. There were also no significant increases in liver or spleen content, although both serum and liver levels were numerically higher for M-Nano-Zn.

The improvement in growth rate of modern broilers needs careful management, to avoid skeletal issues during rearing such as lameness and lameness-related culling, and issues during processing. The results of the current study pinpointed a significant increase in tibia strength associated with feeding the chelated zinc or M-Nano-Zn supplements compared to control at 21 days post hatch. This finding is in alignment with previous studies which indicate zinc that improves bone formation by stimulating cell proliferation, collagen synthesis in osteoblastic cells and mineralisation [40,41,42]. However, by day 35, there were no tibia bone strength differences with only tibia length increased for the nano zinc diets compared to the control. This finding is consistent with a previous zinc supplementation study where no effect on tibia strength in broilers was found at 35 days [43]. Interestingly, [44] observed a positive effect of zinc supplementation only in femurs, but not in tibias at 42 days, suggesting perhaps that zinc accelerates long bone formation but does not ultimately result in increased tibia strength at slaughter age. Therefore, this study indicates that the use of M-Nano-ZnO in broiler diets may decrease the occurrence of skeletal issues during the rearing period. However, the use of M-Nano-ZnO as a dietary supplement may not reduce processing issues associated with leg failures after slaughter.

A concern associated with feeding broilers high zinc diets is that they may go onto to excrete manure with a zinc content higher than the capacity of use by field crops. This in turn may lead to reduction in crop grain yield ([45] in sorghum, [46] in bush beans and maize). In the current study, a significant increase in zinc digestibility was found in birds fed M-Nano-ZnO. This suggests that the environmental impact of zinc supplementation may be mitigated by bioavailable M-Nano-Zn. It should be noted that although both the methionine and the zinc oxide are naturally occurring, the nanomaterial may not be suitable for organic production. This finding has implications in a wider context of mineral supplementation, particularly for minerals such as phosphorous, or for minerals where digestibility is limited.

Spinning disc reactors (SDRs) are becoming widely used in the pharmaceutical and materials industry due to their ability to rapidly perform inorganic and organic reactions under highly controlled conditions while maintaining continuous throughput [47]. Recently, the SDR process has been scaled up in a commercial setting and is currently used to produce horticultural fertilisers for the biofortification of crops such as potatoes and chilis [48]. Due to the high throughput of the SDR (around 8 kg per hour) and the viability of using low cost zinc oxide rather than zinc sulphate, the costs associated with the manufacturing process involved in producing the zinc-methionine nanoparticles is comparable to the combined price of commercial feed grade zinc supplements. Despite this, in some global regions, such as the European Union (EU), legislation currently restricts the use of nanomaterials regardless of their commercial viability [49]. Currently the EU requires all food products to specify whether the material contains engineered nanomaterials [50] and although individual materials are risk assessed for approval on a case by case basis, just 55 are currently registered [51]. However, other regions such as the USA do not legislate the use of nanoparticles in their Food and Drug Administration governance, but provide non-binding recommendations [50]. Whilst safety concerns over the use of nanoparticles in food still exist, these now mainly focus on the unintended ingestion of non-digestible microplastics from packaging and marine fish [52], and within the European Union, the European Food Safety Authority are currently developing a technical framework to facilitate assessment and approval of nanoparticles in food [53].

## 5. Conclusions

A novel method of producing nano minerals coated with amino acids was successfully tested with zinc. Methionine coated nano zinc oxide supplementation significantly improved bird weight gain and increased feed intake of broilers compared to an inorganic zinc form. Ileal digestibility was also improved with this methionine-nano zinc. Moreover, this supplementation improved tibia strength of broilers at the age of 21 days, though this was not observed by day 35. Therefore, M-Nano-ZnO could be used to supplement broilers to improve both performance and digestibility with a limited impact on bone strength.

The results of the current study suggest that amino acid coating of nano minerals can improve the digestibility of minerals which may have further implications for the field of mineral nutrition in animal feeds.

## Figures and Tables

**Table 1:** Ingredient and chemical composition of the basal diets with no added zinc.

	Starter	Grower
Ingredient composition (g/Kg)		
Wheat (10.5% protein)	598.09	646.21
Extracted Soya, (48% protein)	336.79	283.04
Soya oil	32.50	41.82
Limestone	11.20	9.49
Salt	2.02	2.26
Sodium bicarbonate	1.80	1.52
Monocalcium phosphate, HCl	6.48	6.50
Lysine HCl	2.39	1.48
Methionine	3.11	2.41
Threonine	1.42	1.07
Econase XT 25 powder	0.10	0.10
Quantum Blue phytase	0.10	0.10
Zinc-free vitamin/mineral premix *	4.00	4.00
Analysed chemical composition		
Dry matter (g/Kg DM)	874	878
Ash (g/Kg DM)	48.3	52.6
Protein (g/Kg DM)	25.0	21.6
Fat (g/Kg DM)	35.7	60.75
P (g/Kg DM)	7.81	5.13
Ca (g/Kg DM)	9.52	8.13
Gross energy (MJ/Kg DM)	15.9	16

* Premix content (per kg diet): Mn 100 mg, Fe 20 mg, Cu 10 mg, I 1 mg, Mo 0.48 mg, Se 0.2 mg, Retinol 13.5 mg, Cholecalciferol 3 mg, Tocopherol 25 mg, Menadione 5.0 mg, Thiamine 3 mg, Riboflavin 10.0 mg, Pantothenic acid 15 mg, Pyroxidine 3.0 mg, Niacin 60 mg, Cobalamin 30 µg, Folic acid 1.5 mg and Biotin 125 µg; DM = dry matter.

**Table 2:** Effect of zinc type on zinc digested and coefficient of zinc digestibility of broilers.

	Control	M-Zn ^1^	Nano-ZnO ^1^	M-Nano-ZnO ^1^	SEM ^2^	*p* Value
Zn COD ^3^ d21	0.635 ^b,c^	0.685 ^a,b^	0.607 ^c^	0.702 ^a^	0.021	0.003
Zn digested d21 (g/kg)	52.7 ^c^	67.8 ^a^	44.3 ^d^	63.0 ^b^	1.83	<0.001
Zn COD d35	0.662 ^a,b^	0.632 ^b,c^	0.596 ^c^	0.691 ^a^	0.018	0.003
Zn digested d35 (g/kg)	53.6 ^a^	49.8 ^a^	35.2 ^b^	51.1 ^a^	1.40	<0.001

^1^ M-Zn = methionine-Zinc chelate; Nano-ZnO = nano zinc oxide; M-Nano-ZnO = methionine coated nano zinc oxide; ^2^ SEM = standard error of the mean; ^3^ COD = coefficient of digestibility. Superscript letters denote significant differences within rows

**Table 3:** Effect of zinc type on growth performance of broilers.

	Control	M-Zn ^1^	Nano-ZnO ^1^	M-Nano-ZnO ^1^	SEM ^2^	*p* Value
*Starter (d0–21)*						
Bird weight gain (g)	846 ^c^	900 ^b^	914 ^b^	954 ^a^	14.5	<0.001
Feed intake (g)	1156	1182	1205	1214	18.2	0.071
Feed conversion ratio	1.35	1.32	1.33	1.29	0.024	0.375
*Grower (d21–35)*						
Bird weight gain (g)	1341	1343	1372	1397	24.3	0.49
Feed intake (g)	2060	2076	2124	2182	29.6	0.068
Feed conversion ratio	1.53	1.55	1.55	1.56	0.019	0.609
*Overall (d0–35)*						
Bird weight d35 (g)	2232 ^b^	2282 ^a,b^	2324 ^a^	2363 ^a^	33.1	0.044
Bird weight gain (g)	2193 ^b^	2244 ^a,b^	2286 ^a^	2325 ^a^	32.8	0.043
Feed intake (g)	3237 ^b^	3259 ^b^	3312 ^b^	3442 ^a^	40.7	0.006
Feed conversion ratio	1.45	1.45	1.46	1.45	0.013	0.825

^1^ M-Zn = methionine-Zinc chelate; Nano-ZnO = nano zinc oxide; M-Nano-ZnO = methionine coated nano zinc oxide; ^2^ SEM = standard error of the mean. Superscript letters denote significant differences within rows

**Table 4:** Effect of zinc type on tibia length, width, weight and strength of broilers.

	Control	M-Zn ^1^	Nano-ZnO ^1^	M-Nano-ZnO ^1^	SEM ^2^	*p* Value
*Day 21*						
Tibia length (mm)	72.3 ^b^	73.5 ^a^	72.6 ^b^	74.0 ^a^	0.31	<0.001
Tibia weight (g)	7.18 ^c^	7.77 ^a^	7.33 ^b,c^	7.58 ^a,b^	0.12	0.004
Tibia width (mm)	5.46 ^b^	5.74 ^a^	5.45 ^b^	5.60 ^a,b^	0.06	0.001
Tibia strength (N)	181.6 ^b^	197.7 ^a^	178.5 ^b^	188.2 ^a,b^	3.77	0.001
*Day 35*						
Tibia length (mm)	98.3 ^c^	99.1 ^b,c^	100.5 ^a^	99.4 ^a,b^	0.43	0.001
Tibia weight (g)	17.79	17.73	18.15	18.12	0.2	0.365
Tibia width (mm)	7.44	7.47	7.47	7.48	0.06	0.992
Tibia strength (N)	284.2	282.1	298	285.2	5.01	0.084

^1^ M-Zn = methionine-Zinc chelate; Nano-ZnO = nano zinc oxide; M-Nano-ZnO = methionine coated nano zinc oxide; ^2^ SEM = standard error of the mean. Superscript letters denote significant differences within rows.

**Table 5:** Effect of zinc type on zinc content of blood serum (µg/dL), liver and spleen (mg/Kg) of broilers.

	Control	M-Zn ^1^	Nano-ZnO ^1^	M-Nano-ZnO ^1^	SEM ^2^	*p* Value
Serum Zn d21	76.4	73.3	76.8	94.0	8.73	0.404
Serum Zn d35	90.9	106.7	92.3	112.7	8.88	0.067
Liver d21	99.9	112.7	108.5	121.4	10.16	0.133
Liver d35	82.0	83.5	81.7	93.6	4.06	0.173
Spleen d21	92.5	90.6	92.8	93.4	6.23	0.995
Spleen d35	93.3	94.5	92.6	91.8	4.00	0.958

^1^ M-Zn = methionine-Zinc chelate; Nano-ZnO = nano zinc oxide; M-Nano-ZnO = methionine coated nano zinc oxide; ^2^ SEM = standard error of the mean.
